# The Bionic Clicker Mark I & II

**DOI:** 10.3791/55705

**Published:** 2017-08-14

**Authors:** Elliott G. Magee, S. Ourselin, Daniil Nikitichev, T. Vercauteren, Anne Vanhoestenberghe

**Affiliations:** ^1^Aspire Create, University College London; ^2^Translational Imaging Group, CMIC, University College London

**Keywords:** Bioengineering, Issue 126, Electromyography, medical device, public engagement, neuroprosthetic, wireless control, microcontroller, assistive technology, assistive device

## Abstract

In this manuscript, we present two 'Bionic Clicker' systems, the first designed to demonstrate electromyography (EMG) based control systems for educational purposes and the second for research purposes. EMG based control systems pick up electrical signals generated by muscle activation and use these as inputs for controllers. EMG controllers are widely used in prosthetics to control limbs.

The Mark I (MK I) clicker allows the wearer to change the slide of a presentation by raising their index finger. It is built around a microcontroller and a bio-signals shield. It generated a lot of interest from both the public and research community.

The Mark II (MK II) device presented here was designed to be a cheaper, sleeker, and more customizable system that can be easily modified and directly transmit EMG data. It is built using a wireless capable microcontroller and a muscle sensor.

**Figure:**
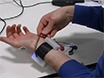


## Introduction

The Aspire Centre for Rehabilitation Engineering and Assistive Technology investigates techniques that are applicable and transferable between different domains in related areas of interest, including but not limited to, stroke, muscular dystrophy, amputation, the ageing population and training of specialized skills. One area of research that the center is involved in is neuroprosthesis. Of the many techniques used for control of neuroprosthetic arms, EMG is one of the most common inputs for the control systems^1^,^2^,^3^,^4^,^5^,^6^. This is in a large part due to its ease of use and affordability when compared to other control systems^7^. Recently developed 3D-printed prostheses such as the Ada hand can cost only 1,000 USD when using this type of control^8^,^9^,^10^. However, when attempting to demonstrate such systems to the public there is no easy way of doing so without the aid of an amputee.

To raise the awareness of the research activities in this field to members of the public, a bionic clicker demo device was developed. It is very important to use object-based demonstration as it attracts attention and accelerates the learning and understanding of the subject being taught^11^. Our device not only helps to teach the concept of EMG but also to increase the knowledge of the current development of modern technologies. Moreover, it inspires younger generations to choose studies within Science, Technology, Engineering and Math (STEM) areas.

The Bionic Clicker MK I was made using readily available parts that were already in use within the lab. It consisted of a microcontroller, a bio-signals shield^12^, electrodes, a control board, a wireless communication board and a 9 V battery. The device functioned by picking up the activity of the abductor indicis muscle located between the index finger and thumb. It triggers a slide change by mimicking a keyboard and sending a 'right keypress' whenever a preselected threshold was reached. The control board allowed for the manual sending of 'right' and 'left' keypresses (progress slides and retreat slides) and could also be used to override the EMG input if things went wrong during a live demonstration.

As part of the Medical Physics and Biomedical Engineering department public engagement activities, we demonstrated the Bionic Clicker to members of the public. It received an enthusiastic response from the audience and generated interest in starting several collaborations. After the success of the initial device a second version of the device was designed.

The goal for the design of the second device was to produce a device that was cheaper, less cumbersome and more customizable than the first device. The purpose of this device was to design something that could easily be modified for student projects and be cheaply incorporated into existing projects. The main advantage of this device over others available^1^,^2^,^3^,^4^,^5^,^6^ is its ease of use, small size, and low cost. Although the bionic clicker devices may not have the resolution of other research devices, such as trigger devices, they are more than good enough. The MK II would be an ideal basis for any system that uses an EMG threshold to trigger a device, such as a prosthetic controller or assistive device.

The design was based around a wireless-capable microcontroller and a muscle sensor. It also included a 3.7 V 150 mAh lithium polymer battery, a manual control board and a 3D-printed case. **Figure 3** shows an overview of the differences between the designs. The MK II design has the same basic functionality as the original device but has significantly more potential functionality for new applications such as wireless EMG monitoring.

## Protocol

The protocol follows the guidelines of the University College London's human research ethics committee.

CAUTION: This protocol contains an electrical hazard and a burn hazard (soldering iron); read both before attempting this protocol. This protocol includes connecting a device to skin. Ensure that at no time there is a path between the skin and electricity mains. Never touch the element of the soldering iron. Hold wires to be heated with tweezers or clamps. Keep the cleaning sponge wet during use. Always return the soldering iron to its stand when not in use. Never put it down on the workbench. Turn unit off and unplug it when not in use.

### 1. Assembling the Base Components

NOTE: Figure 3 gives a high-level overview of the protocol steps.

1. To build the Bionic Clicker MK I, plug the bio-signals sensor shield into the microcontroller and screw the EMG cables into the E, M and GND screw terminals of the shield (see Figure 4). Continue to step 1.6.
2. **To build the MK II, place a row of three header pins into the muscle sensor in the +, - and SIG holes (see Figure 5) from above, and solder underneath.**
  1. Bend the header pins 90 ° with a pair of plyers halfway up the pins. This places the pins in the correct position for the case.
  2. If using the abductor indicis muscle as the input, continue to step 1.3, if not move to step 1.4.
3. **Remove the short black reference cable from the muscle sensor.**
  1. Cut the three EMG cables with a wire cutter to run from the wrist to the back of the hand. Strip the end of the three EMG cables with a wire stripper.
  2. Place the stripped end of the black wire into the R hole, the blue wire into the E hole, and the red wire into the M hole of the muscle sensor (Figure 5). Solder the wires in place on the underside of the muscle sensor. Move to step 1.5.
4. **Clip two electrode pads into the underside of the muscle sensor and one electrode into the connector of the black reference cable.**
  1. Stick the muscle sensor onto the selected muscle with the electrode pads and place the black reference electrode in an appropriate place.
5. **Cut 8 single-core multi-thread wires to length and strip each end: 5 short (7 cm) wires to run from the microcontroller to the control board (red, black, green, white and blue) and 3 longer (approximately 12 cm but dependent on wrist size) wires (red, black and green) to run from one side of the wrist to the other.** NOTE: If placing the muscle sensor on a different muscle make sure that the longer wires will run from the muscle sensor site to the wireless microcontroller site.
  1. Place the wires into the microcontroller ready for soldering: 2 red wires into the 3V hole, 2 black wires into the GND hole, the long green wire into the A0 hole, the short blue wire into the 2 hole, the long white wire into the 3 hole, and the short green wire into the 5 hole. Solder the wires in place on the underside of the microcontroller.
  2. Solder the other end of the 3 long wires to 3 header sockets in the order: red, black, green. See Figure 5. If not using the abductor indicis muscle proceed on to step 2.
6. **Place the EMG sensor pads on the hand, as shown in Figure 6, with two of the electrodes at either end of the abductor indicis muscle and one EMG sensor pad on the middle of the back of the hand.**
  1. Clip the electrode pads into the connector end of the muscle sensor cables (push fit). The blue and red electrodes clip above the muscle, the black electrode clips on the back of the hand.

### 2. Test EMG Output

1. Download the library for the bio-signals shield following the link^14^ from the reference section. Unzip it and place it in the Integrated Development (IDE) libraries folder (usually found in documents/Arduino/libraries). Continue to step 2.3. If building the MK II, proceed to step 2.2.
2. Add the microcontroller boards to the IDE, following the instructions^15^.
3. Download 'ThresholdTest.ino' for the MK I or 'BLEThresholdTest.ino' and 'BluefruitConfig.h' for the MK II and open in the IDE software (Supplemental files).
4. Unplug the laptop from the mains and then, and only then, plug the microcontroller into the laptop via a Universal Serial Bus (USB) cable.
5. Upload the relevant version of the threshold test to the microcontroller and then open the serial monitor (Tools>Serial Monitor). The output of the EMG will now be displayed.
6. Move the index finger from side to side and move the hand around without moving the index finger. Write down the values displayed in each case. NOTE: When using the MK II make sure that the cables do not move as it is extremely sensitive to noise generated this way.
7. Select a value that is above what is seen when the hand is moved around, but below what is seen when the finger is moved from side to side. Write down this value. NOTE: The value is selected so that the device will only be activated by a purposeful movement of the finger. This is the Threshold Trigger Value, the value at which the device will be activated. The muscle sensor has a gain setting that can be manually altered if the threshold value is hard to find. The electrodes might need to be replaced. If using the abductor indicis muscle, set the gain to minimum as the starting point. The gain setting is altered by the potentiometer on the muscle sensor marked by GAIN, and this can be changed by a small flat-head screwdriver.

### 3. Test Threshold

1. Download 'BoomTest.ino' for the MK I or 'BLEBoomTest.ino' and BluefruitConfig.h for the MK II and open it in the IDE software.
2. Edit the provided code by replacing 'PLACE_YOUR_THRESHOLD_TRIGGER_VALUE_HERE' with the Threshold Trigger Value previously determined in step 2.8. This is line 37 of the code for the MK I and line 47 of the code for the MK II.
3. Upload the correct version of BoomTest to the microcontroller and then open the serial monitor (Tools>Serial Monitor).
4. Move the hand around (not moving the index finger from side to side); nothing is seen on the serial output.
5. Move the index finger from side to side; the word 'BOOM' appears. NOTE: If 'BOOM' appears at the wrong time or not at all, check the connections and move back to step 2.7.

### 4. 3D Print the MK II Case

1. If building the MK II, download the stl files for all 5 components of the case (see Figure 7 for all 5 parts). Print the parts of the case by any preferred method. Proceed to step 5.2. If building the MK I, move on to section 5. NOTE: The case has been successfully printed by both fused deposition modelling^16^ (FDM) and photolithography printers^17^.

### 5. Solder the Control Board

NOTE: If building the MK II, proceed to step 5.2.

1. **Place a row of two header pins, five 10 KΩ resistors, a sliding switch and two push button switches for the components as shown in Figure 8A; then solder them in place on the underside of the board.**
  1. Break the copper tracks on the strip board by slicing through with a craft knife, following the grey lines on Figure 8**A**. This allows for individual tracks to have multiple functions across the board.
  2. Cut 7 wires (black, red, blue, orange, white, brown and yellow) of the correct length with a wire cutter so that they will run from the forearm to the upper arm (around 30 cm). Cut a red wire of 7 cm, a black wire of 3 cm and an orange and a blue wire of 4 cm.
  3. Strip both ends of the wires with a wire stripper.
  4. Place the wires in the control board, following the circuit diagram shown in Figure 9; solder the wires in place on the underside.
  5. Solder the long red and black wires to a pair of header pins, and then solder the other long wires to a strip of header pins in the order: blue, orange, white, brown, yellow.
  6. Solder the 5V and GND pins of the wireless module to the header pins on the control board.
  7. Solder the short orange wire to pin 2 of the wireless communication module and the short blue wire to pin 3.
2. **Place three 10 KΩ resistors, a sliding switch and two push button switches as shown in Figure 10A and solder them into place on the underside of the board.**
  1. Break the copper tracks on the strip board by slicing through with a craft knife, following the grey lines on **Figure 10A**. This allows for the track to have multiple functions on the board.
  2. Cut the wires that were previously soldered to the microcontroller with a wire cutter so that they can run through the mid layer of the microcontroller case to the control board without stopping the case from closing (Figure 10**B**).
  3. Place the wires in the control board, following the circuit diagram (Figure 11). Solder the wires in place. Proceed to step 6.2.

### 6. Assemble Clicker and Update Microcontroller

1. Re-assemble the Bionic Clicker, connecting the header connectors from the control board wires to the microcontroller and bio-signals shield (5V and GND on the MK I, pin 22-30 on the MKII). Connect the battery to the microcontroller. See Figure 12. Move on to step 6.3.
2. Re-assemble the Bionic Clicker, connecting the header connector from the microcontroller to the muscle sensor (green wire to SIG). See Figure 13.
3. Connect the microcontroller to the laptop via USB cable.
4. Download 'BionicClicker.ino' or 'BLEBionicClicker.ino and BluefruitConfig.h and open it in the IDE software.
5. Edit the code and replace 'PLACE_YOUR_THRESHOLD_TRIGGER_VALUE_HERE' with the Threshold Trigger Value determined in step 2.7 (on line 59 of the code for the MK I, line 83 of the code for the MK II). NOTE: The name that the MK II device appears as when connecting over wireless can be changed by editing **line 47** of the code. Replace '**Bionic Clicker MK II**' with an alternative title.
6. Disconnect the microcontroller from the laptop by removing the USB cable.

### 7. Connect the Device to a Computer

1. If using the MK I, follow the instructions to pair the wireless module to the device by following the manufacturer's guide^18^. If using the MK II, connect to the device wirelessly following the procedure to connect a wireless keyboard to the computer being used.

### 8. Test the Clicker

1. **Open some typing software and enter some text, such as 'Lorem ipsum dolor sit amet'. This allows the presses to be perceived to test whether these commands are sent and received.** NOTE: If the battery is low the device may give erratic behavior; always use a fresh battery.
  1. Press the manual forward button to see the cursor move forward and the manual backwards button to see the cursor move backwards. Raise the index finger to also move forward.
2. To test the clicker with the presentation software, raise the index finger to progress the slides. NOTE: The override switch turns the EMG function on and off, and the manual forward and backwards buttons progress and retreat the slides in both scenarios.

### 9. Mount the Clicker

NOTE: If building the MK II move to step 9.2.

1. **If building the MK I, cut the double-sided hook and loop material with scissors, so that it fits comfortably around the wrist. Make sure the loops are facing inwards to not scratch the wrist.**
  1. Cut the double-sided hook and loop material so that it fits comfortably around the upper arm, again make sure the loops face inwards.
  2. Cut the double-sided hook and loop strips to the size of the microcontroller (10 cm x 5 cm) and the control board (2.5 cm x 6.4 cm). Cut a strip that will fit tightly around the battery (4 cm x 12 cm).
  3. Using the glue gun, glue the loop side of the strips to the bottom of the microcontroller and the bottom of the control board.
  4. Attach the control board to the wrist strap. Attach the microcontroller and battery to the upper arm strap.
  5. Plug everything in: The 9 V battery plugs into the microcontroller with the PP3 connector. The microcontroller and e-health shield connect to the control board via the soldered wires. NOTE: The MK I is now finished.
2. **If building the MK II, cut double-sided hook and loop material 35 mm wide and long enough to wrap around the wrist (around 22 cm for smaller wrists).**
  1. Slide the hook and loop material through the clips at the bottom of the case. Make sure that the loops are facing inwards to not scratch the wrist.
  2. Plug the wires soldered to the microcontroller terminating in the female header into the male header pins on the muscle sensor, and clip the electrodes into the EMG cables by pushing them on. NOTE: The MK II is now finished. See Figure 14.

## Representative Results

The MK II is more affordable, customizable and less cumbersome than the MK I device. The entire MK II costs only slightly more than the bio-signals shield alone (75 USD). The device is significantly smaller sitting on the wrist rather than the arm and the wireless microcontroller could potentially simultaneously support inputs from 6 muscle sensors. The functional battery life of the MK I device is just under an hour using a 9 V 550 mAh battery and the functional battery life of the MK II device (when used as a clicker) is around 8 hours using a 3.7 V 150 mAh battery; see **Table 1** for a comparison of the devices.

The Bionic Clicker MK II can have an issue when used on the abductor indicis: the amplifier can saturate and take more than a second to discharge (see Figure 15). Careful placement of the electrodes and correctly setting the gain can overcome this issue. This does not happen with the Bionic Clicker MK I or on any other commonly used muscles for EMG.

Whilst calibrating the devices to find the Threshold Trigger Value, many different values can be observed. They fall into three ranges: the values when the hand is stationary, the values when the hand is moving, and the values when the finger is moved. **Table 2** shows recorded values in each range; for the stationary and hand moving ranges, the maximum values are shown and for the finger tensing range the minimum value is shown. The threshold value is selected to lie above the hand moving value and below the finger tensing value. A value nearer to the hand moving range increases the chance of false positive and reduces the chance of false negatives, whilst a value closer to the finger tensing range has the opposite effect.

Both devices where tested for false negatives and false positives when tensing the abductor indices muscle. A false negative was recorded when the device did not trigger a change of slide upon tensing of the muscle and a false positive was recorded if the slide changed when no tensing occurred. Neither device had an issue with false positives, though the MK II device experienced the occasional false negative (less than 5% of the time). The MK I device experiences no false positives or negatives during the first 45 minutes of operation, though the number of false negatives increases rapidly until total device failure between 50 minutes and an hour (see **Table 3**).

These results show that the device succeeded in its stated aims. **Table 1** shows that the MK II is cheaper and has more flexibility than the MK I. **Table 2** and **Table 3** show that the device functions as intended and can be used as an EMG-based trigger device. Figure 15 shows the issues that can occur if using the abductor indices muscle: this is not a problem that occurs with most muscles and can be fixed by altering the gain. Although the devices have some issues, they are sufficient for the intended use.

**Figure:**
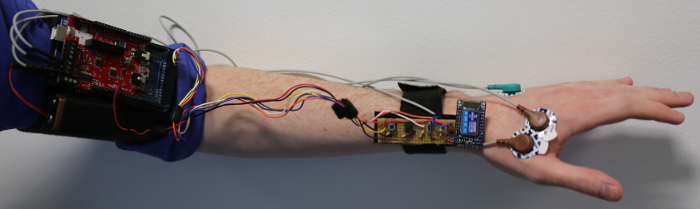

Figure 1:** The Bionic Clicker MK I. **This shows the Bionic Clicker MK I and all of its components mounted on the left arm. Please click here to view a larger version of this figure.

**Figure:**
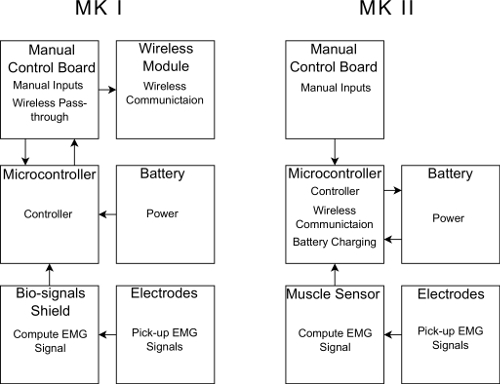

Figure 2:** Block diagram of the devices.** Each box represents a separate section of the device; within each box is the functionality that section has as a part of the device. Please click here to view a larger version of this figure.

**Figure:**
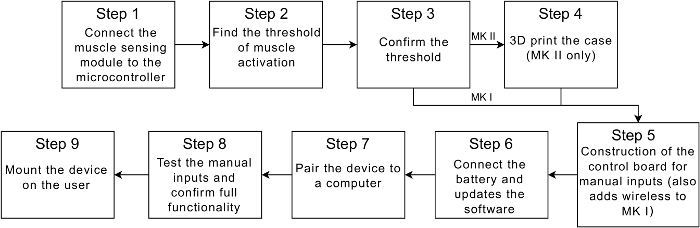

Figure 3**: Steps to build the device.** A flow diagram containing a high-level overview of each step of the protocol. Please click here to view a larger version of this figure.

**Figure:**
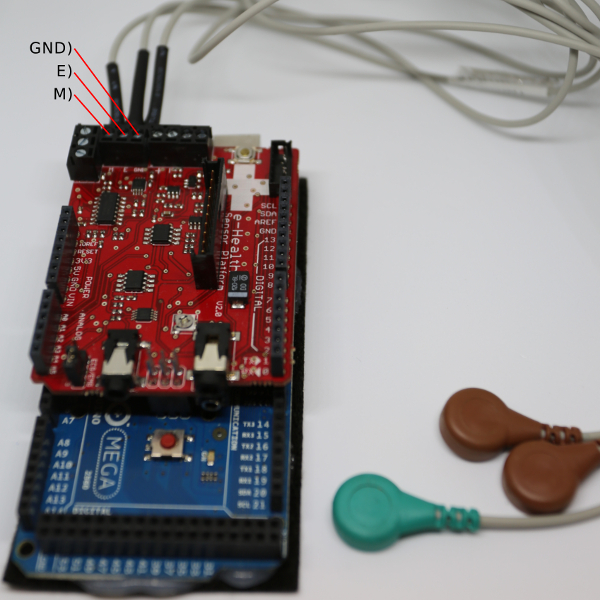

Figure 4**: Initial MK I assembly.** Microcontroller with the bio-signals shield and electrode cables. Please click here to view a larger version of this figure.

**Figure:**
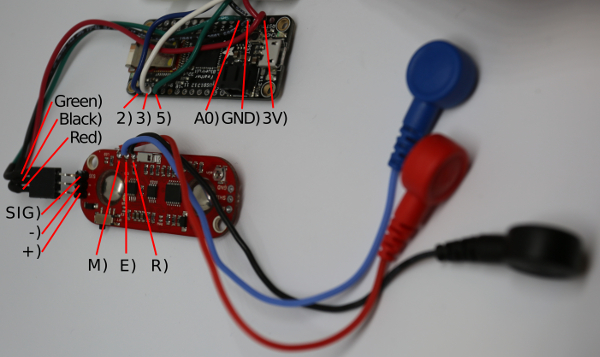

Figure 5:** Initial MK II assembly.** Microcontroller with the muscle sensor and soldered connections. Please click here to view a larger version of this figure.

**Figure:**
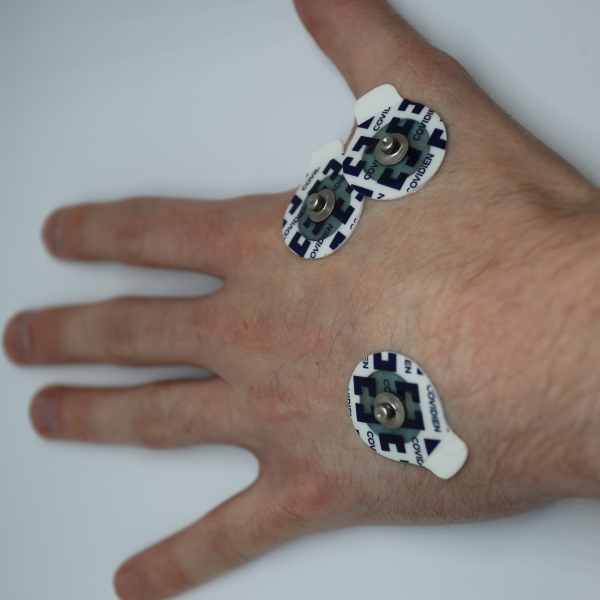

Figure 6**: Electrode Placement.** This figure shows the correct placement of the electrodes on the hand when using the abductor indicis. Please click here to view a larger version of this figure.

**Figure:**
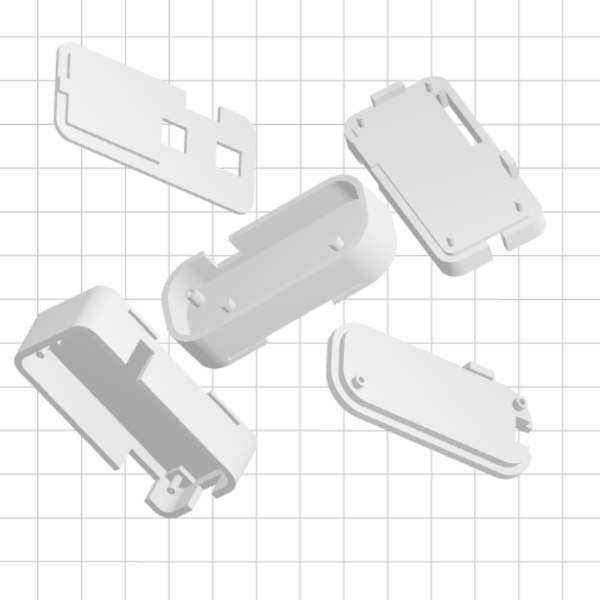

Figure 7**: The MK II case parts.** The parts of the MK II case ready for printing in a photolithography printer. Please click here to view a larger version of this figure.

**Figure:**
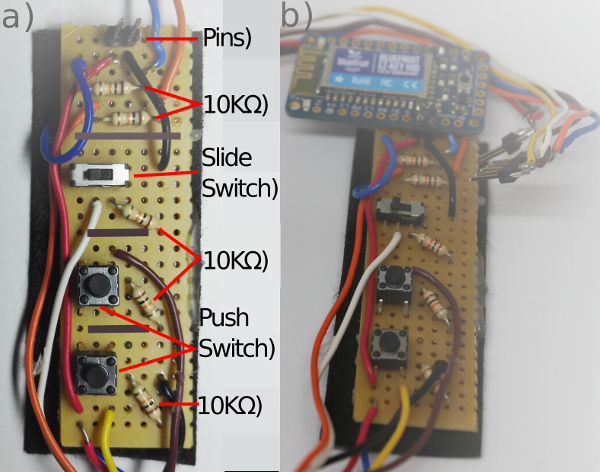

Figure 8**: The MK I control circuit.** (**a**) Circuit board from above (gray marks where the strip board had contacts broken on the underside). (**b**) Completed Circuit Board. Please click here to view a larger version of this figure.

**Figure:**
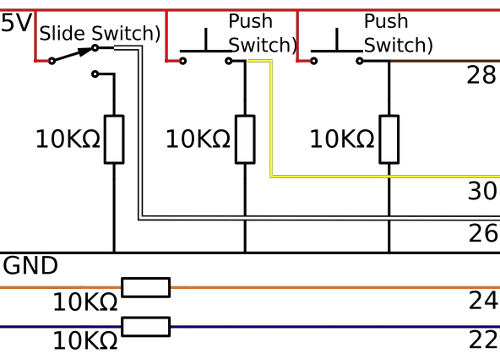

Figure 9**: The MK I control board circuit diagram.** The circuit diagram for the MK I control board showing the connections between the resistors, switches and wires. Please click here to view a larger version of this figure.

**Figure:**
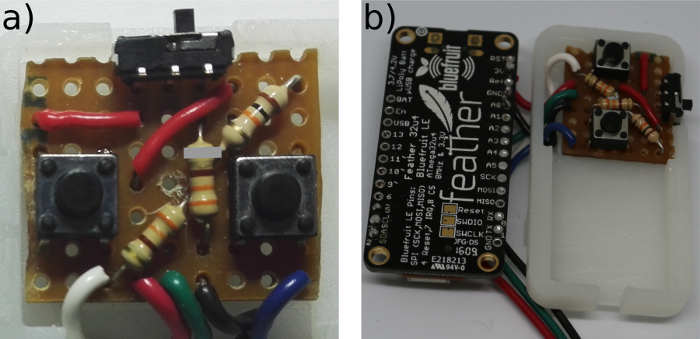

Figure 10**: The MK I control circuit.** (**a**) Control Board from above (gray mark where the strip board had contact broken on the underside). (**b**) Completed Circuit Board Please click here to view a larger version of this figure.

**Figure:**
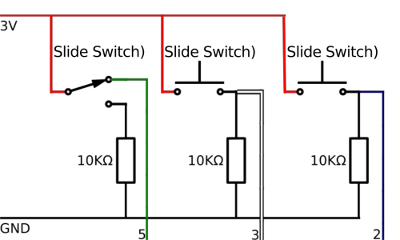

Figure 11:** The MK II control board circuit diagram.** The circuit diagram for the MK I control board showing the connections between the resistors, switches and wires. Please click here to view a larger version of this figure.

**Figure:**
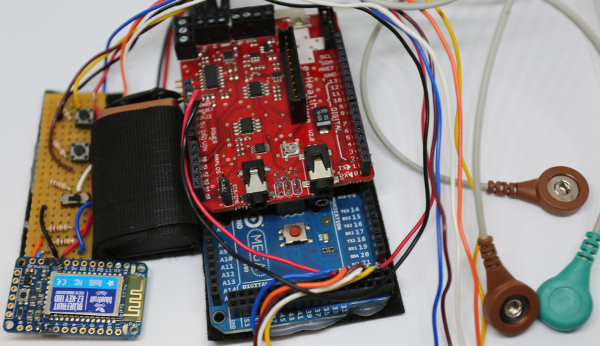

Figure 12**: The assembled MK I.** This shows all the components of the MK I device before they have been mounted on the arm. Please click here to view a larger version of this figure.

**Figure:**
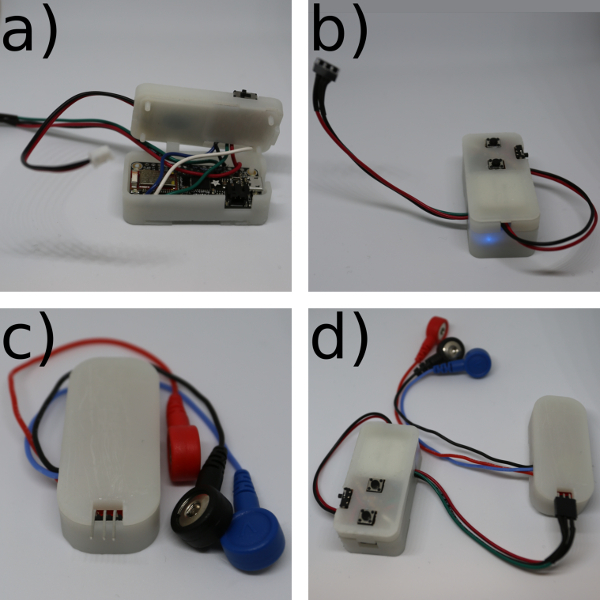

Figure 13**: Assembling the Clicker MK II.** (**a**) Place the microcontroller in the bottom of the case. (**b**) Place the battery in the mid-section and put on the lid. (**c**) Place the muscle sensor in its case and put on the lid. (**d**) Connect the microcontroller to the muscle sensor and connect the battery to the microcontroller. Please click here to view a larger version of this figure.

**Figure:**
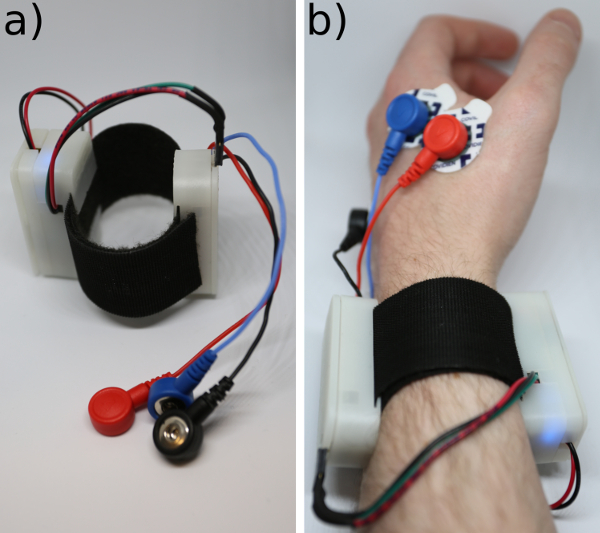

Figure 14**: The completed Bionic Clicker MK II.** (**a**) On the hook and loop strap. (**b**) On the wrist. Please click here to view a larger version of this figure.

**Figure:**
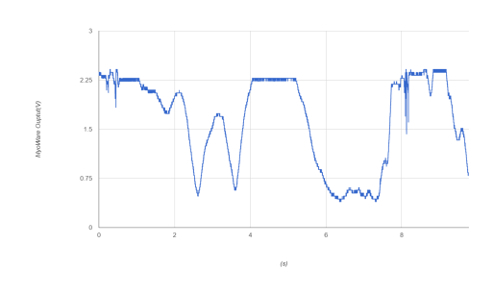

Figure 15:** Oversaturation of the muscle sensor. **This figure shows what happens when the muscle sensor is oversaturated; the plateaus are when muscle activation was too strong for the current gain setting on the device. Please click here to view a larger version of this figure.

**Table:** 

	**MK I**	**MK II**
**EMG sensor**	General Bio-sensor	Dedicated Muscle Sensor
**Wireless**	Separate wireless module	On the microcontroller board
**EMG over wireless?**	No	Yes
**Battery**	9 V PP3	150 mAh LiPo
**Operational Time**	1 h	8 h
**Build Time**	5 h	4 h
**Total cost**	$150	$80
**False Positives (%)**	0	0
**False Negatives (%)**	0	4.7

**Table 1: Comparison of the devices**. This table compares several aspects of the devices, from design to functionality.

**Table:** 

	**Stationary (maximum)**	**Hand Moving (maximum)**	**Finger Tensing (minimum)**	**Threshold Value**
**MK I**	25	35	215	200
**MK II**	40	280	460	400

**Table 2: Calibration Results. **This table shows the values obtained whilst keeping the hand stationary, moving the hand and finger tensing, as well as the threshold value selected.

**Table:** 

	**Number of false negatives (Tested every 30 s)**	**Number of false positives (Spontaneous activations)**
	**First 45 min**	**45 min-1 h**	**1-8 h**	**First h**	**1-8 h**
**MK I**	0	35	N/A	0	N/A
**MK II**	4	1	40	0	0

**Table 3: Testing of the devices**. Comparison of false positives and false negatives between the two devices.

**Supplemental code files for MK I and MK II:**Please click here to download "BionicClicker.ino"Please click here to download "BLEBionicClicker.ino".Please click here to download "BLEBoomTest.ino".Please click here to download "BLEThresholdTest.ino".Please click here to download "BoomTest.ino".Please click here to download "ThresholdTest.ino".Please click here to download "Feather-Featherbase.stl".Please click here to download "Feather-Feathermid.stl".Please click here to download "Feather-Feathertop.stl".Please click here to download "Myo-Myobase.stl".Please click here to download "Myo-Myolid.stl".  

## Discussion

The saturation of the MK II when used on the abductor indicis is less of an issue than it may first appear. Careful placement of the electrodes and correct gain setting stops this from being an issue when the device is used as a clicker. Unless interested in accurately recording the activity of the abductor indices, this is unlikely to be a problem at all. No over-saturation has been seen on any other muscle after the gain has been set. The false negatives with the MK II are due to the difficulty of selecting the proper threshold value when using the abductor indicis. With larger muscles the difference between the magnitude of non-purposeful activation of the muscle and purposeful tensing of the muscle is greater, allowing for the selection of a threshold point that is further from both the false-positive and false-negative points. On particularly small hands the abductor indicis muscle may be too small for the electrodes to be correctly placed (though with smaller electrode pads this could potentially be solved).

The considerably longer battery life for the MK II is useful for a variety of reasons. Firstly, the MK I device started to act erratically after 45 minutes of use, so it cannot be used for longer demonstrations. Secondly with a multi-hour battery life, the MK II can be considered as a control input for a useful device, and with only a small increase in physical battery size, it could be used as an all-day monitoring device. The wireless microcontroller has 6 analogue inputs and 13 digital inputs; this means that the device could accept signals from multiple muscle sensors to create a device with more degrees of freedom in the control inputs. It should also be noted that the muscle sensor could be replaced by any biosensor with an analogue output to create a device that uses other biological signals as the input. The code of the device can also be easily modified to change its functionality. Changes to the software and hardware of the device allow for simple and varied modifications to the device.

One limitation of the device as it currently stands is that the EMG output cannot be sent wirelessly at a high data rate as this can overload the wireless microcontroller buffer. Another limitation is that the technique uses the abductor indices as the input, and as the muscle is very small, the spacing of the electrodes on the hand almost overlap; if someone has particularly small hands, it may be impossible to place the electrodes correctly over this muscle. 

The device has several advantages over the more expensive devices when it comes to flexibility in potential research projects. It is low cost: the device costs 80 USD and additional EMG channels only cost 35 USD, making it ideal for smaller or student projects. It is easy to customize, the software can be easily edited, and the inputs changed for other hardware. It has a small size, so a person wearing it does not need to carry heavy or bulky equipment. It also appears as a wireless keyboard to other devices, so it can be easily integrated with any compatible wireless device. The device has already been incorporated into an assistive device that will be published in the near-future.

Due to the size and ease of customization of the MK II, it is already being considered for incorporation into several research projects as a wireless EMG module and as a wireless trigger mechanism. It is also being used as the foundation of one of the lab sessions on a master student's course. The main improvement we would like to make to the device is to increase the wireless transmission rate; the goal is to achieve 10 Hz, and whether this will be done through hardware or software is yet to be determined. 

The most critical steps within the protocol are steps 2.6 and 2.7: the selection of the Threshold Trigger Value. In step 2.6, special attention needs to be paid to the movement of the EMG cables, as these can act as antenna and generate motion artifacts; however, if these are kept stationary this is not an issue. In step 2.7, if the selected value is too high, this results in false negatives. If this value is too low, this results in false positives. In the case of the abductor indicis, it is very difficult to find a value that does not result in the occasional false negative, although with larger muscles this does not appear to be an issue. If finding the correct value is still an issue, the gain can be corrected by setting it to the minimum value and increasing it until a large difference between non-activation and activation is seen through the serial readout, with the values staying below the point of saturation.

Overall the MK II is a considerable improvement over the MK I as a potential research device, although because the MK I has a stronger visual impact, it is likely to still be used in future public engagement events.

## Disclosures

The authors have nothing to disclose.
